# Global profiling of α-glucosidase inhibitors from *Citri Reticulatae Pericarpium* based on affinity ultrafiltration screening coupled with UPLC-ESI-Orbitrap-MS method

**DOI:** 10.1371/journal.pone.0340990

**Published:** 2026-01-13

**Authors:** Hongping Wang, Qiong Yin, Zhaozhou Lin, Quantao Ma, Zhaohua Zhang, Jun Jia

**Affiliations:** 1 Department of Drug Screening and Development, Scientific Research Institute of Beijing Tongrentang Co., Ltd., Beijing, China; 2 Department of Drug Screening and Development, Beijing Tongrentang Technology Development Co., Ltd., Beijing, China; Hong Kong Baptist University, HONG KONG

## Abstract

As a medicinal and edible herb, *Citri Reticulatae Pericarpium* has multiple biological activities. The latest modern pharmacological researches have found that *Citri Reticulatae Pericarpium* has the effect of lowering blood sugar, and its extract can inhibit the activity of α-glucosidase. However, which component has inhibitory activity on α-glucosidase and the degree of inhibition are not clear. In order to solve this problem, this study used affinity ultrafiltration screening coupled with UPLC-ESI-Orbitrap-MS method to systematically screen α-glucosidase inhibitors from *Citri Reticulatae Pericarpium* for the first time. Through affinity ultrafiltration technology, the active components were selected, and through UPLC-ESI-Orbitrap-MS technology, their structures were identified. Finally, total 84 active ingredients were selected as α-glucosidase inhibitors, and most of them were multimethoxy flavonoids. Our results indicated that inhibiting α-glucosidase activity was probably one of the most important mechanisms for *Citri Reticulatae Pericarpium* exerting its hypoglycemic effect. In addition, our results first reported that multimethoxy flavonoids had the effects of hypoglycemic activity and potential anti-diabetes value.

## Introduction

*Citri Reticulatae Pericarpium* is the dried and mature peel of the Rutaceae plant *Citrus reticulata* Blanco and its cultivated varieties, with a history of over 2000 years of application in China. In ancient China, *Citri Reticulatae Pericarpium* was commonly used for treatment of various syndromes including Digestive symptoms: Spleen and stomach qi stagnation; abdominal distension, nausea, vomiting, and lack of appetite caused by cold and dampness in gastric cavity; Respiratory symptoms: Coughing and excessive phlegm due to phlegm dampness obstructing the lungs [1], because it has the effects of regulating qi and strengthening the spleen, drying dampness and resolving phlegm. Regarding the development of pharmacological effects, modern researchers have found that *Citri Reticulatae Pericarpium* has a range of pharmacological effects including

1) Anti-inflammatory [2–3], reducing inflammation and alleviating symptoms associated a variety of different diseases.
2) Neuroprotective: Treating Alzheimer’s disease and Parkinson’s disease [4–5].
3) Anti-cancer [6–7], inhibiting the growth of cancer cells and preventing tumor progression.
4) Bone protection [8–9], supporting bone health and preventing bone-related disorders.
5) Anti-obesity [10–11], aiding in weight management and reducing the risk of obesity-related diseases.
6) Skin protection [12–13], protecting the skin from damage and promoting overall skin health.
7) Vascular regulation [14–15], supporting cardiovascular health and regulating blood vessel function. Therefore, the application of *Citri Reticulatae Pericarpium* in modern clinical practice not only follows the ancient usage, but also expands into new application areas to treat respiratory diseases such as chronic obstructive pulmonary disease, bronchiectasis, and elderly asthma; cardiovascular diseases such as coronary heart disease, angina pectoris, heart failure; digestive system diseases such as chronic gastritis, irritable bowel syndrome; advanced gastric cancer; stroke and sequelae. In addition, *Citri Reticulatae Pericarpium* is also clinically used as an auxiliary treatment for diabetes [16]. However, the hypoglycemic mechanism and the active ingredients of *Citri Reticulatae Pericarpium* in treating of diabetes are not well understood, which has become a basic scientific issue needing to be solved urgently so as to meet the requirements of evidence-based medicine.

α-Glucosidase is one of the important targets for diabetes, and inhibition of the activity of α-glucosidase can slow down carbohydrate digestion and glucose absorption. Commonly used α-glucosidase inhibitors include: Acarbose, voglibose, and miglitol are the commonly used α-glucosidase inhibitors in clinical practice. Based on α-glucosidase target, some researchers explored the mechanism of *Citri Reticulatae Pericarpium* for treating diabetes, and finally found that *Citri Reticulatae Pericarpium* extract had a certain inhibitory effect on α-glucosidase [17], indicating *Citri Reticulatae Pericarpium* probably could exert hypoglycemic effects by inhibiting the activity of α-glucosidase. However, which component has inhibitory activity on α-glucosidase and the degree of inhibition are unclear, and in order to solve this problem, this study screened α-glucosidase inhibitors from *Citri Reticulatae Pericarpium*.

The traditional methods for screening active ingredients from traditional Chinese medicine are mainly based on multiple extractions and separations, and there are mainly two approaches. One is activity oriented separation, which uses *in vitro* pharmacological evaluation to evaluate the activity of each stage of extraction and separation, track its active components, and then continue to track the significantly active components until the active monomer components are obtained [18]. The other is to directly extract, separate, and structurally identify the chemical components in traditional Chinese medicine, and then perform biological activity determination to determine the active ingredients [19]. The two research strategies mentioned above often have long experimental cycles and large workloads, making it impossible to achieve large-scale and high-throughput screening. With the development of active ingredient screening technology, researchers have found that screening active ingredients in traditional Chinese medicine based on drug targets can improve screening efficiency, and at the same time, a series of research methods have been developed, such as cell membrane chromatography (CMC) [20–21], magnetic bead enrichment technology [22–23], surface plasmon resonance (SPR) [24–25] as well as affinity ultrafiltration coupled with ultra-performance liquid chromatography-high resolution mass chromatography (UPLC-HR-MS) [26–27]. Compared with traditional active ingredient screening methods, these screening techniques exhibit the advantages of high efficiency and high-throughput screening. However, the first three techniques require the target protein to be fixed on a specific carrier, which has special and high requirements for the carrier. Moreover, the target protein fixation process is cumbersome and can easily lead to enzyme inactivation. Affinity ultrafiltration coupled with UPLC-HR-MS technology directly co-incubates targets with traditional Chinese medicine extracts to maximize the retention of enzyme activity, which is simpler to operate than the first three methods. Through affinity ultrafiltration technology, active ingredients can be directly captured, and through high-resolution mass spectrometry technology, active ingredients can be quickly identified. Therefore, affinity ultrafiltration coupled with UPLC-ESI-Orbitrap-MS was used to select α-glucosidase inhibitors from *Citri Reticulatae Pericarpium.* As a result, total 84 α-glucosidase inhibitors were selected, and most of them were multimethoxy flavonoids. Our study systematically screened and identified α-glucosidase inhibitors from *Citri Reticulatae Pericarpium* for the first time, and the results suggested that inhibiting α-glucosidase activity was probably one of the most important mechanisms for *Citri Reticulatae Pericarpium* exerting its hypoglycemic effect.

## Materials and methods

Enzymes: – α-Glucosidase from *Saccharomyces cerevisiae* (with purity ≥50%) used for affinity ultrafiltration screening was obtained from Sigma (Enzyme Commission number: 3.2.1.20, 100 units, MO, USA), whereas α-glucosidase from *Saccharomyces cerevisiae* used for the detection of the equilibrium dissociation constants (Kd) value was purchased from MCE (No. HY-P2802, 100 units, NJ, USA) based on its purity ≥90%, which meets the experimental requirements.

Buffers and Reagents: – Phosphate buffer (0.1M, pH 6.8) and ammonium acetate buffer (10 mM, pH 6.86) were obtained from Applygen Technologies Inc (Beijing, China). *p*-Nitrophenyl-α-D-glucopyranoside (pNPG) were purchased from Macklin Biochemical Technology Co., Ltd (Shanghai, China). The distilled water was obtained from Watsons. - Sodium carbonate (0.1M) and DMSO were purchased from Sigma (MO, USA). - LC-MS-grade acetonitrile and methanol were obtained from Merck (Darmstadt, Germany), whereas - LC-MS-grade formic acid was purchased from Fisher-Scientific (Fair Lawn, NJ, USA).

Kits and Other Materials: – Biotinylation kit was obtained from Beijing Sankeshi Chuangyuan Technology Co., Ltd. (Beijing, China), and PBS buffer (pH 7.4) was purchased from Gibco (NY, USA).

Plant Material: *Citri Reticulatae Pericarpium* was supplied by the Scientific Research Institute of Beijing Tongrentang Co., Ltd., and identified by Prof. Xiu-Wei Yang, who is from the State Key Laboratory of Natural and Biomimetic Drugs, School of Pharmaceutical Sciences, Peking University, China. A voucher specimen (no. 202202) has been deposited in the Scientific Research Institute of Beijing Tongrentang Co., Ltd. (Beijing, China).

Compounds: A total of 22 compounds, including acarbose, 5-hydroxy-3,7,3’,4’-tetramethoxyflavone (**C1**), 5-demethylnobiletin (**C2**), vicenin-3 (**C3**), naringin (**C4**), 8-hydroxy-3,5,6,7,3′,4′-hexamethoxyflavone (**C5**), 4′,5,6,7-tetramethoxyflavone (**C6**), tangeretin (**C7**), neohesperidin (**C8**), nobiletin (**C9**), quercetagetin-3,5,6,7,3′,4′-hexamethyl ether (**C10**), sinensetin (**C11**), isosinensetin (**C12**), hesperidin (**C13**), naringenin (**C14**), naringenin-7-O-glucoside (**C15**), eriocitrin (**C16**), vicenin-2 (**C17**), rhoifolin (**C18**), hesperetin (**C19**), isoquercitroside (**C20**), and kaempferol-3-O-rutinoside (**C60**) were all purchased from Shanghai Yuanye Bio-Technology Co., Ltd. (Shanghai, China). The purity of all the reference standards was > 98%.

### Sample preparations

*Citri Reticulatae Pericarpium* was pulverized into a fine powder (the size was just like flour). The powder of *Citri Reticulatae Pericarpium* (10 g) was ultrasonically extracted for 30 min with 100 mL 70% methanol at 25 °C. The extracted solution was filtered through a filter paper. And then, the extraction process was repeated twice. The filtrate was combined and evaporated to dryness (4.4 g) using a rotary evaporator at 40 °C. The residue (10 mg) was then dissolved in 1 mL of ammonium acetate buffer (10 mM, pH 6.86) and filtered through a 0.22-μm nylon filter membrane to obtain the sample of affinity ultrafiltration screening.

### Affinity ultrafiltration screening

The affinity ultrafiltration procedure was performed according to our previously reported method [26]. The freeze-dried powder of α-glucosidase was dissolved in 10 mM ammonium acetate buffer (pH 6.86) to obtain α-glucosidase solution (40 U/mL). A total of 100 μL *Citri Reticulatae Pericarpium* sample solution (10 mg/mL) was incubated with 100 μL α-Glucosidase (40 U/mL) for 30 min at 37 °C. And then, the incubation solution was transferred into an ultrafiltration centrifugal filter (AMICON ULTRA, 0.5 mL, 10 kDa, Millipore, Massachusetts, USA) containing a regenerated cellulose ultrafiltration membrane with a 10,000 MW cut-off at 25 °C for centrifuge (14,000 r/min for 10 min). The retained α-glucosidase–ligands complexes were washed five times using 250 μL ammonium acetate buffer (pH 6.86) each time followed by centrifuge (14,000 r/min for 10 min) to remove the unbound compounds. After that, α-glucosidase–ligands complexes were dissociated three times using 100 μL of methanol–water (50:50; *v*/*v*, pH 3.30) each time to release the bound ligands, followed by centrifuge (14,000 r/min for 15 min). All of the dissociation solution was combined and evaporated to dryness using a nitrogen-blowing instrument, and then 50 μL methanol–water (50:50; *v*/*v*) was added to re-dissolve the residue. The control experiment was carried out with denatured enzyme (in boiling water for 10 min) instead of the active enzyme. Each pair of sample and control specimens was prepared in four replicates. The obtained re-dissolved solution was then analyzed by UPLC-ESI-Orbitrap-MS.

### UPLC-ESI-Orbitrap-MS analysis

A Vanquish™ Flex UPLC system (Thermo Scientific, Massachusetts, USA) equipped with a binary pump and a thermostated column compartment was used to analyze the sample and control specimens, and a Waters ACQUITY UPLC^®^ BEH C_18_ column (2.1 × 100 mm, 1.7 μm) (Waters, Milford, USA) coupled with an ACQUITY UPLC^®^ BEH C_18_ VanGuard^TM^ Pre-Column (2.1 × 5 mm, 1.7 μm) was used to separate the components using mobile phase A (0.1% formic acid/water, *v*/*v*) and mobile phase B (acetonitrile) by the following gradient elution program: 0–7 min, 2–20% B; 7–10 min, 20–25% B; 10–20 min, 25–40% B; 20–25 min, 40–65% B; 25–30 min, 65–95% B. The flow rate was 0.3 mL/min and the injection volume was 2 μL. The temperature was set at 35 °C.

An Orbitrap Exploris 240 mass spectrometer (Thermo Scientific, Massachusetts, USA) equipped with a Heated ESI source was used to acquire the mass data in positive-ion mode. The MS parameters were set as follows: ion spray voltage: 3400 V, sheath gas: 5.08 L/min, auxiliary gas: 9.37 L/min, ion transfer tube temperature: 320 °C, vaporizer temperature: 350 °C, scan range (*m*/*z*): 150–1000, and collision-energy (%): 30, 45, 60. An internal calibration source, Thermo Scientific EASY-IC^TM^ (Thermo Scientific, Massachusetts, USA), was adopted to calibrate the entire mass range. The full scan was operated at a mass resolution of 60,000 whereas the MS^2^ scan was operated at a mass resolution of 15,000.

### Molecular docking study

The 2D structures of the selected ligands were downloaded from PubChem, and their 3D structures were generated in Chemdraw Ultra (version 14.0). Due to the 3D structure of α-glucosidase from *Saccharomyces cerevisiae* is not available in Protein Data Bank (PDB), the 3D structure of isomaltase (PDB code: 3A4A), which is with 84% similarity to *Saccharomyces cerevisiae* α-glucosidase [28], was downloaded for molecular docking. More information about 3A4A was detailed as UniProt ID: P53051; classification: hydrolase; organism(s): *Saccharomyces cerevisiae*; expression system: escherichia coli; mutation(s): no; deposited: 2009-07-01; released: 2010-07-14; method: X-ray diffraction; resolution: 1.60 Å; R-value free: 0.174 (Depositor), 0.170 (DCC); R-value work: 0.159 (depositor), 0.160 (DCC); R-value observed: 0.159 (depositor); predicted active sites through CavityPlus (http://repharma.pku.edu.cn/cavityplus): VAL-232, SER-240, PHE-303, ASP-307, VAL-308, ASP-242, VAL-319, ASP-409, ASP-352, ASN-350, PRO-354, LEU-313, GLU-332, PRO-243, PHE-178, VAL-109, THR-306, ASN-247, TRP-155, THR-245, PHE-321, PRO-312, ASP-233, PRO-230, PHE-159, ARG-442, LEU-219, TRP-238, HIS-280, MET-70, HIS-351, GLN-353, PRO-320, TYR-316, ASP-325, GLN-239, GLN-182, ARG-446, LEU-177, ARG-213, TYR-72, SER-241, TRP-326, SER-236, TYR-347, ASP-73, VAL-216, ASP-69, LEU-246, PHE-314, SER-304, ALA-281, SER-311, GLY-161, ASN-302, LYS-156, ILE-440, GLU-411, GLY-309, ASN-415, ARG-315, ALA-329, HIS-112, GLU-277, GLN-279, THR-310, ASP-107, SER-157, HIS-305, ASP-215, CYS-179, TYR-158, and GLY-160. The 3A4A structure was pre-processed by removing solvent and adding hydrogen atoms. AutoDock Tools (version 1.5.6) was used to predict the affinitie values of 3A4A and ligands. The grid box was set as follows: number of points in x-dimention: 40; number of points in y-dimention: 40; number of points in z-dimention: 40; spacing 1.0; for center grid box, x center: 21.276; y center: −0.752; z center: 18.634. The structural cartoons were prepared using PyMOL (version 2.4.0).

### Molecular dynamics simulation

After molecular docking, 200 ns molecular dynamics simulations were performed using Gromacs (2023.2) to evaluate the binding stability of the α-glucosidase-ligand complexes. CHARMM 36 was the protein force field, whereas GAFF was the small molecule force field. The SPCE model was used to add TIP3P water solvent to the system, establishing a water box with a size of 16.118 × 16.118 × 16.118 nm, and add ions to automatically balance the system charge. In the process of molecular dynamics simulation, the steepest descent method was first used as the initial step for energy minimization, with a maximum of 10000 iterations. And then, the conjugate gradient method was used for further energy minimization, with a maximum of 10000 iterations. The convergence criterion was set to stop iteration when the energy change was less than 1000 kJ/mol/nm. Subsequently, 100000 steps of isothermal-isobaric-isochoric (NVT) ensemble equilibrium and normal pressure and temperature (NPT) ensemble equilibrium were performed, with a time step of 2 fs and a duration of 2000 ps for each stage. After minimizing and balancing the system energy, molecular dynamics simulations were conducted for TNF and CASP3 at 100 ns, while Eadd and CASP8 at 200 ns without any constraints. Finally, we analyzed the root mean square deviation (RMSD), root mean square fluctuation (RMSF), radius of gyration (Rg), solvent accessible surface area (SASA), the number of hydrogen bonds between α-glucosidase and ligands, and the distribution of free energy in the molecular dynamics simulation trajectories of each complex.

### Detection of the Kd value and the α-g*lucosidase inhibitory activity assay*

The Kd value was detected using Octet^®^ R8 (Sartorius, Germany) equipped with Octet^®^ SSA Biosensors (Part Number 18–5057). α-Glucosidase was dissolved in PBS solution to obtain the α-glucosidase solution (1 mg/mL). A total of 0.1 mL Tween 20 was added into 500 mL PBS solution to obtain PBST solution. α-Glucosidase was first incubated with 10 mM biotinylation reagent to obtain biotinylated α-glucosidase, and then solidified. Appropriate amount of each tested compound was separately dissolved in DMSO to obtain the stock solution (6 mM), and then appropriate amount of stock solution was diluted using PBST + 0.5% DMSO to obtain six sample solutions in different concentrations (30 μM, 10.0 μM, 3.0 μM, 1.0 μM, 0.3 μM, and 0.1 μM). A total of 200 μL PBST + 0.5% DMSO was added to the buffer well of the sample plate, whereas 200 μL of different concentrations of sample solutions were added to the sample well. By setting up a detection program, the affinities of compounds and α-glucosidase were detected.

The α-glucosidase inhibitory activity assay was performed in 96-well plates according to the modified method of Li [29]. Both pNPG and α-glucosidase were separately dissolved in a 0.1 M phosphate buffer (pH 6.8). Appropriate amount of each tested compound was first dissolved in 50 μL of DMSO to obtained its stock solution, and then 0.1 M phosphate buffer was added to give solutions of various concentrations. A total of 40 μL of the tested compound solution was incubated with 40 μL α-glucosidase solution (0.2 U/mL) at 37 °C for 5 min, and then 20 μL of pNPG solution (2 mM), which was used as a substrate, was added in the incubation solution After incubating at 37 °C for 30 min, total 100 μL of sodium carbonate (0.1M) was added to terminate the reaction, and then the amount of released nitrophenyl product was measured on an Epoch 2 microplate spectrophotometer (BioTek) at 405 nm. Acarbose was used as the positive control. Controls contained the same reaction mixture, except the same volume of 0.1 M phosphate buffer (pH 6.8) was added instead of a solution of tested compounds. The inhibition (%) of the tested ligands on α-glucosidase was calculated as: (A_a_ − A_b_)/A_a_ × 100%, where A_a_ was the absorbance of the control, and A_b_ was the absorbance of the tested compound.

## Results and discussion

### Selection of α-glucosidase inhibitors by affinity ultrafiltration screening-UPLC-ESI-Orbitrap-MS technology

**Fig 1:**
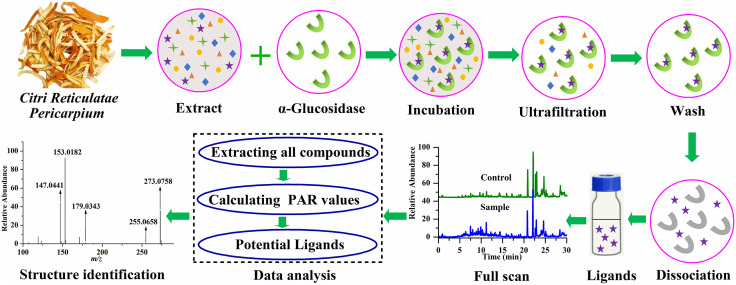
The workflow of selection of α-glucosidase inhibitors form *Citri Reticulatae Pericarpium.*

The workflow of selection of α-glucosidase inhibitors (shown in Fig 1) was clarified briefly as follows: the *Citri Reticulatae Pericarpium* extract was incubated with α-glucosidase, and active ligands bound to the active site of α-glucosidase forming receptor–ligands complexes, whereas the unbound compounds were free. The incubation solution was then transferred into an ultrafiltration centrifuge tube containing an ultrafiltration membrane to remove the unbound compounds by washing and centrifuging multiple times. The receptor–ligands complexes were disrupted by adding dissociation agent, and the released ligands were analyzed by UPLC-ESI-Orbitrap-MS in a full scan mode. The obtained data was systematically analyzed to obtain the active ligands, and then their structures were characterization through fragmentation ions.

**Fig 2:**
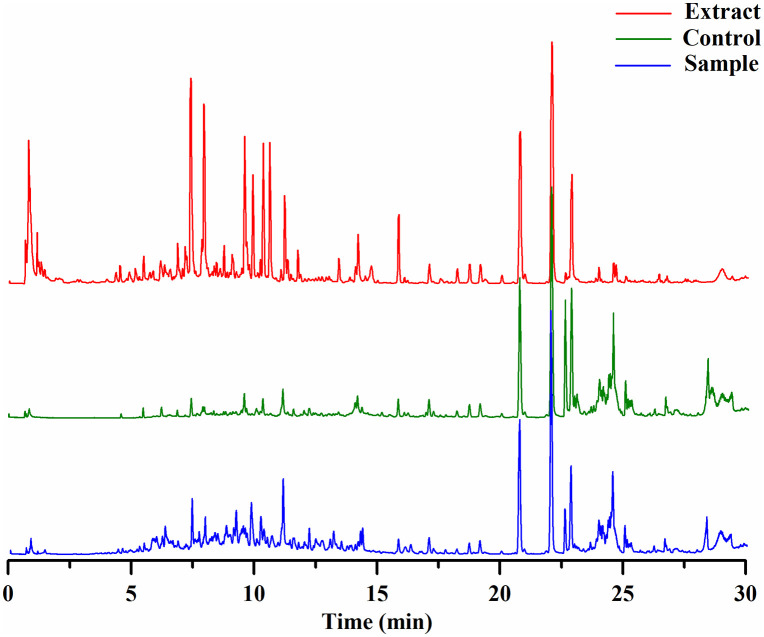
The total ion chromatograms of specimens. The extract of *Citri Reticulatae Pericarpium* (red color); the control group (green color), and the sample group (blue color).

The total ion chromatograms of *Citri Reticulatae Pericarpium* extract, the control and sample specimens were shown in Fig 2. From Fig 2, we found compounds in control and sample specimens were complicate, and even some peaks were overlapped, in this case, directly comparing the intensity of each peak in their total ion chromatograms between the sample and the control groups could lead to false positive or false negative results. To improve the accuracy of screening, we established an effective data process workflow described as follows:

(1) Extracting all compounds in each specimen

The sample and control specimens were first detected in a full scan mode, and the obtained data were imported into Compound Discoverer^TM^ software (Thermo Scientific^TM^, version 3.2.0.421) for data analysis. All compounds in the data of sample and control specimens were extracted and aligned based on the extracted workflow “input files → select spectra → align retention time → detect compounds → group compounds”. In the “input files” step, the obtained full MS data were imported, whereas in the “select spectra” step, the spectrum properties such as retention time for data processing (0–30 min) and the polarity mode (positive) were set. In the “align retention time” step, all compounds were aligned (mass tolerance: 5 ppm), whereas in the “detect compounds” step, they were extracted by setting a series of parameters, such as mass tolerance: 5 ppm; intensity tolerance: 30; S/N threshold: 3; minimum peak intensity: 100,000; extracted ions: [M + H]^+^; minimum element composition: CHO, and maximum element composition: C_90_H_190_O_90_. In the “group compounds” step, the same compounds detected in different addition ways were grouped according to their molecular weight (mass tolerance: 5 ppm) and retention time (RT tolerance: 0.1 min). Each extracted compound was presents by its molecular weight, retention time, and peak area, *etc.*

(2) Calculating the peak area ration of each compound and t-test

When the peak areas of each compound in sample and control specimens were separately obtained, we began to calculate its peak area ratio (PAR), which was defined as the peak area ratio of a compound in the sample group to the blank group. And then, their mean PAR was calculated. The significant difference in peak area of each compound between the sample and the control group was determined by a two-tailed t-test. The compound with mean PAR value >1 and *p *< 0.05 (*n* = 4) were selected as the potential α-glucosidase inhibitors.

(3) Characterizing the structures of ligands

The precursor ions of the potential α-glucosidase inhibitors as well as their retention times were imported into a precursor ion list for targeted MS/MS analysis, and the obtain fragmentation ions were used for structures identification.

**Fig 3:**
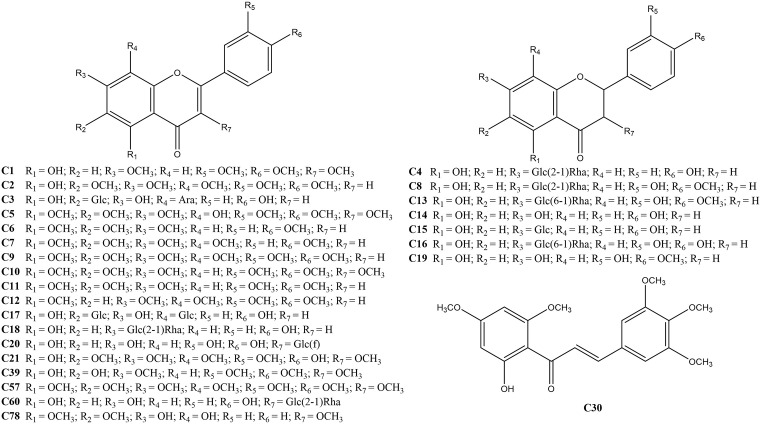
The selected ligands with definite structures.

By running the established data process, total 84 flavonoids (shown in Table 1), including 26 known compounds (shown in Fig 3) and 58 unknown compounds which were the isomers of the known compounds, were successfully selected, and the data of affinity ultrafiltration screening was shown in Supplementary Information S1 Table. It was worth noting that in our preliminary research [30], we had comprehensively characterized the chemical components in the extract of *Citri Reticulatae Pericarpium*, which provided convenience for the structural inference of the active ligands screened in this experiment. Although a total of 187 chemical ingredients were identified in *Citri Reticulatae Pericarpium* in the previous study [30], this study found that only 84 of these ingredients had inhibitory activity against α-glucosidase. The identified ligands and their fragmentation ions were shown in Supplementary Information S2 Table, and among them, compounds **C1**–**C20** were confirmed by comparison with their reference standards.

**Table 1:** The α-glucosidase inhibitors selected from *Citri Reticulatae Pericarpium* extract.

No.	*t*_R_ (min)	Molecular formula	Detected MW	PAR value	Compound name
Compounds of reference standards commercially available					
**C1**	25.09	C_19_H_18_O_7_	358.1052	1.99 ± 0.05	5-Hydroxy-3,7,3’,4’-tetramethoxyflavone
**C2**	23.81	C_20_H_20_O_8_	388.1157	1.83 ± 0.18	5-Demethylnobiletin
**C3**	7.98	C_26_H_28_O_14_	564.1478	1.69 ± 0.15	Vicenin-3
**C4**	9.92	C_27_H_32_O_14_	580.1789	1.68 ± 0.01	Naringin
**C5**	24.62	C_21_H_22_O_9_	418.1263	1.67 ± 0.04	8-Hydroxy-3,5,6,7,3′,4′-hexamethoxyflavone
**C6**	20.93	C_19_H_18_O_6_	342.1102	1.62 ± 0.16	4′,5,6,7-Tetramethoxyflavone
**C7**	22.82	C_20_H_20_O_7_	372.1206	1.61 ± 0.21	Tangeretin
**C8**	10.73	C_28_H_34_O_15_	610.1903	1.57 ± 0.29	Neohesperidin
**C9**	20.73	C_21_H_22_O_8_	402.1311	1.51 ± 0.28	Nobiletin
**C10**	20.00	C_21_H_22_O_8_	402.1313	1.47 ± 0.07	Quercetagetin-3,5,6,7,3′,4′-hexamethyl ether
**C11**	18.69	C_20_H_20_O_7_	372.1207	1.41 ± 0.23	Sinensetin
**C12**	17.05	C_20_H_20_O_7_	372.1207	1.40 ± 0.14	Isosinensetin
**C13**	10.32	C_28_H_34_O_15_	610.1894	1.39 ± 0.09	Hesperidin
**C14**	10.03	C_15_H_12_O_5_	272.0685	1.35 ± 0.09	Naringenin
**C15**	10.06	C_21_H_22_O_10_	434.1213	1.32 ± 0.03	Naringenin-7-O-glucoside
**C16**	8.55	C_27_H_32_O_15_	596.1741	1.32 ± 0.11	Eriocitrin
**C17**	6.86	C_27_H_30_O_15_	594.1584	1.27 ± 0.19	Vicenin-2
**C18**	9.58	C_27_H_30_O_14_	578.1635	1.23 ± 0.17	Rhoifolin
**C19**	15.57	C_16_H_14_O_6_	302.0790	1.19 ± 0.08	Hesperetin
**C20**	8.72	C_21_H_20_O_12_	464.0954	1.18 ± 0.03	Isoquercitroside
Compounds of reference standards commercially unavailable					
**C21**	23.72	C_20_H_20_O_9_	404.1107	2.31 ± 0.02	5,4´-Dihydroxy-3,6,7,8,3´-pentamethoxyflavone
**C22**	6.46	C_27_H_32_O_15_	596.1741	2.29 ± 0.70	Eriocitrin isomer
**C23**	12.61	C_19_H_18_O_7_	358.1047	2.18 ± 0.10	5-Hydroxy-3,7,3’,4’-tetramethoxyflavone isomer
**C24**	24.16	C_20_H_20_O_8_	388.1157	2.10 ± 0.55	5-Demethylnobiletin isomer
**C25**	23.19	C_19_H_18_O_7_	358.1053	2.02 ± 0.09	5-Hydroxy-3,7,3’,4’-tetramethoxyflavone isomer
**C26**	23.03	C_20_H_22_O_7_	374.1368	1.98 ± 0.78	2´-Hydroxy-3,4,4´,5´,6´-pentamethoxychalcone isomer
**C27**	16.18	C_20_H_22_O_7_	374.1365	1.37 ± 0.02	2´-Hydroxy-3,4,4´,5´,6´-pentamethoxychalcone isomer
**C28**	9.93	C_28_H_34_O_15_	610.1903	1.92 ± 0.20	Neohesperidin isomer/Hesperidin isomer
**C29**	22.32	C_19_H_18_O_7_	358.1053	1.79 ± 0.07	5-Hydroxy-3,7,3’,4’-tetramethoxyflavone isomer
**C30**	19.38	C_20_H_22_O_7_	374.1366	1.76 ± 0.10	2´-Hydroxy-3,4,4´,5´,6´-pentamethoxychalcone
**C31**	7.18	C_15_H_12_O_5_	272.0684	1.75 ± 0.23	Naringenin isomer
**C32**	8.43	C_21_H_20_O_12_	464.0955	1.74 ± 0.17	Isoquercitroside isomer
**C33**	8.55	C_21_H_22_O_10_	434.1213	1.74 ± 0.11	Naringenin-7-O-glucoside isomer
**C34**	10.17	C_15_H_12_O_5_	272.0684	1.73 ± 0.12	Naringenin isomer
**C35**	14.16	C_21_H_22_O_9_	418.1264	1.69 ± 0.05	8-Hydroxy-3,5,6,7,3′,4′-hexamethoxyflavone isomer
**C36**	9.10	C_27_H_30_O_14_	578.0000	1.69 ± 0.24	Rhoifolin isomer
**C37**	23.48	C_20_H_20_O_8_	388.1158	1.65 ± 0.04	5-Demethylnobiletin isomer
**C38**	10.72	C_16_H_14_O_6_	302.0790	1.64 ± 0.22	Hesperetin isomer
**C39**	21.06	C_19_H_18_O_8_	374.1001	1.63 ± 0.34	Quercetagetin-3,7,3′,4′-tetramethyl ether
**C40**	16.79	C_20_H_20_O_8_	388.1158	1.58 ± 0.21	5-Demethylnobiletin isomer
**C41**	19.03	C_18_H_16_O_7_	344.0896	1.58 ± 0.06	Dihydroxy-trimethoxyflavone isomer
**C42**	8.41	C_27_H_30_O_14_	578.0000	1.57 ± 0.31	Rhoifolin isomer
**C43**	15.81	C_21_H_22_O_9_	418.1264	1.57 ± 0.15	8-Hydroxy-3,5,6,7,3′,4′-hexamethoxyflavone isomer
**C44**	8.41	C_26_H_28_O_14_	564.1478	1.55 ± 0.17	Vicenin-3 isomer
**C45**	8.51	C_15_H_12_O_5_	272.0684	1.53 ± 0.24	Naringenin isomer
**C46**	22.39	C_20_H_20_O_9_	404.1107	1.53 ± 0.06	5,4´-Dihydroxy-3,6,7,8,3´-pentamethoxyflavone isomer
**C47**	9.26	C_27_H_30_O_15_	594.1585	1.53 ± 0.14	Kaempferol-3-O-rutinoside isomer
**C48**	22.09	C_20_H_20_O_9_	404.1107	1.52 ± 0.16	5,4´-dihydroxy-3,6,7,8,3´-pentamethoxyflavone isomer
**C49**	20.41	C_18_H_16_O_7_	344.0896	1.52 ± 0.08	Dihydroxy-trimethoxyflavone isomer
**C50**	8.26	C_28_H_34_O_15_	610.1899	1.50 ± 0.18	Neohesperidin isomer/Hesperidin isomer
**C51**	15.97	C_20_H_20_O_8_	388.1157	1.49 ± 0.04	5-Demethylnobiletin isomer
**C52**	17.79	C_21_H_22_O_9_	418.1263	1.49 ± 0.09	8-Hydroxy-3,5,6,7,3′,4′-hexamethoxyflavone isomer
**C53**	12.02	C_27_H_30_O_14_	578.0000	1.48 ± 0.26	Rhoifolin isomer
**C54**	7.30	C_27_H_32_O_15_	596.1741	1.48 ± 0.21	Eriocitrin isomer
**C55**	15.20	C_27_H_32_O_14_	580.1792	1.46 ± 0.13	Naringin isomer
**C56**	14.97	C_19_H_18_O_7_	358.1052	1.46 ± 0.26	5-Hydroxy-3,7,3’,4’-tetramethoxyflavone isomer
**C57**	22.02	C_22_H_24_O_9_	432.1416	1.45 ± 0.08	3,3′,4′,5,6,7,8-heptamethoxyflavone
**C58**	18.59	C_21_H_22_O_9_	418.1263	1.45 ± 0.07	8-Hydroxy-3,5,6,7,3′,4′-hexamethoxyflavone isomer
**C59**	23.93	C_21_H_22_O_8_	402.1313	1.44 ± 0.18	Quercetagetin-3,5,6,7,3′,4′-hexamethyl ether isomer/Nobiletin isomer
**C60**	8.64	C_27_H_30_O_15_	594.1585	1.44 ± 0.09	Kaempferol-3-O-rutinoside
**C61**	19.33	C_20_H_20_O_7_	372.1210	1.42 ± 0.09	Tangeretin isomer/Isosinensetin isomer/Sinensetin isomer
**C62**	8.25	C_26_H_28_O_14_	564.1479	1.41 ± 0.16	Vicenin-3 isomer
**C63**	8.26	C_16_H_14_O_6_	302.0790	1.39 ± 0.20	Hesperetin isomer
**C64**	17.59	C_20_H_20_O_8_	388.1157	1.39 ± 0.21	5-Demethylnobiletin isomer
**C65**	19.02	C_19_H_18_O_7_	358.1053	1.39 ± 0.20	5-Hydroxy-3,7,3’,4’-tetramethoxyflavone isomer
**C66**	9.56	C_21_H_22_O_10_	434.1212	1.38 ± 0.18	Naringenin-7-O-glucoside isomer
**C67**	20.06	C_18_H_16_O_7_	344.0896	1.38 ± 0.04	Dihydroxy-trimethoxyflavone isomer
**C68**	18.19	C_21_H_22_O_8_	402.1313	1.37 ± 0.25	Quercetagetin-3,5,6,7,3′,4′-hexamethyl ether isomer/Nobiletin isomer
**C69**	20.70	C_20_H_20_O_7_	372.1240	1.37 ± 0.02	Tangeretin isomer/Isosinensetin isomer/Sinensetin isomer
**C70**	16.00	C_19_H_18_O_7_	358.1052	1.36 ± 0.28	5-Hydroxy-3,7,3’,4’-tetramethoxyflavone isomer
**C71**	9.57	C_27_H_32_O_14_	580.1789	1.35 ± 0.16	Naringin isomer
**C72**	14.14	C_19_H_18_O_7_	358.1053	135 ± 0.02	5-Hydroxy-3,7,3’,4’-tetramethoxyflavone isomer
**C73**	10.30	C_27_H_32_O_15_	596.1741	1.35 ± 0.10	Eriocitrin isomer
**C74**	18.15	C_19_H_18_O_6_	342.1103	1.35 ± 0.17	4′,5,6,7-Tetramethoxyflavone isomer
**C75**	10.32	C_16_H_14_O_6_	302.0790	1.34 ± 0.07	Hesperetin isomer
**C76**	17.05	C_19_H_18_O_7_	358.1047	1.34 ± 0.06	5-Hydroxy-3,7,3’,4’-tetramethoxyflavone isomer
**C77**	9.57	C_15_H_12_O_5_	272.0684	1.33 ± 0.17	Naringenin isomer
**C78**	10.31	C_18_H_16_O_7_	344.0896	1.28 ± 0.05	Dihydroxy-trimethoxyflavone
**C79**	19.12	C_19_H_18_O_6_	342.1101	1.27 ± 0.14	4′,5,6,7-Tetramethoxyflavone isomer
**C80**	7.39	C_27_H_32_O_14_	580.1790	1.25 ± 0.20	Naringin isomer
**C81**	7.39	C_21_H_22_O_10_	434.1213	1.25 ± 0.15	Naringenin-7-O-glucoside isomer
**C82**	14.53	C_19_H_18_O_7_	358.1053	1.24 ± 0.09	5-Hydroxy-3,7,3’,4’-tetramethoxyflavone isomer
**C83**	8.25	C_15_H_12_O_5_	272.0684	1.19 ± 0.14	Naringenin isomer
**C84**	7.39	C_15_H_12_O_5_	272.0685	1.15 ± 0.10	Naringenin isomer

**Fig 4:**
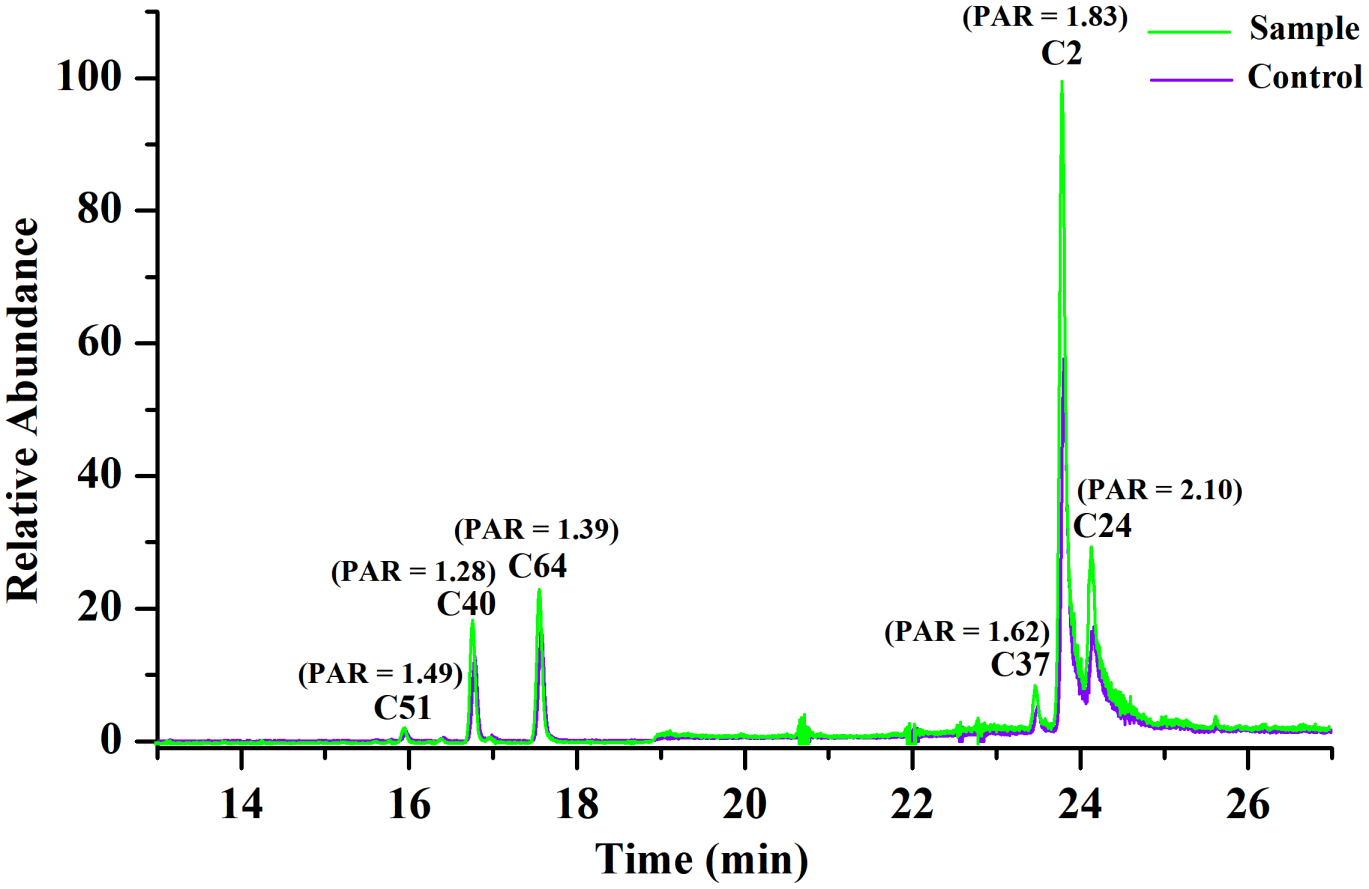
The PAR values of some representative ligands.

In addition, in order to confirm that there was indeed a difference in peak intensity between the sample and the control group for the selected ligands, we separately extracted their ion chromatograms in the sample and control specimens, and we found all of the selected ligands exhibiting higher peak intensities in the sample group, and the PAR values as well as the peak intensity of some representative ligands were shown in Fig 4.

### Molecular docking study

**Fig 5:**
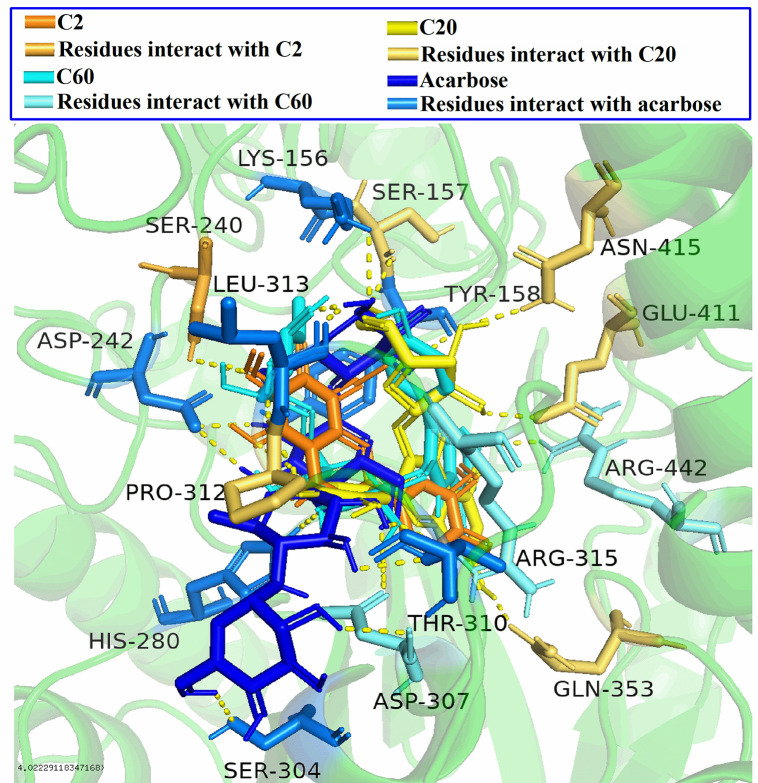
The interaction between representative compounds and 3A4A.

The molecular docking model was first tested using the positive control acarbose (a widely used α-glucosidase inhibitor), and the affinity value was −8.6 kcal/mol (shown in Table 2). This indicated that acarbose had a moderate affinity with 3A4A, and also demonstrated the reliability of the molecular docking process used in this study. Next, this molecular docking process was used to predict the interaction between the screened compounds and 3A4A. Fig 5 showed the interaction between representative compounds and 3A4A. 5-Demethylnobiletin (**C2**) interacted with residue SER-240 through one hydrogen bond, and isoquercitroside (**C20**) interacted with residues SER-157, PRO-312, GLN-353, GLU-411 as well as ASN-415 through five hydrogen bonds. Kaempferol-3-O-rutinoside (**C60**) interacted with residues ASP-307, PRO-312, ARG-315, and ARG-442 through four hydrogen bonds, whereas acarbose interacted with residues LYS-156, TYR-158, ASP-242, HIS-280, SER-304, ASP-307, THR-310, and LEU-313 through ten hydrogen bonds. The residues of 3A4A interacted with the other compounds were shown in Table 2. The residues LYS-13, LYS-156, SER-157, TYR-158, ASP-215, SER-240, ASP-242, GLU-277, GLN-279, HIS-280, ASP-307, THR-310, PRO-312, ARG-315,ASP-352, GLN-353, GLU-411, ASN-415, and ARG-442 were considered as very important active sites for 3A4A, because they interacted with at least two selected compounds through hydrogen bonds. In addition, compared with the positive control drug acarbose, the screened compounds except **C1**, **C2**, **C6**, **C11**, **C12**, **C17**, **C20**, **C21**, **C39** as well as **C57**, all interact with 3A4A through at least one identical residue. It is worth noting that molecular docking sites of the selected compounds with 3A4A all fall within the predicted active site range, except LYS-13, LYS-16 and ASN-259. Additionally, literature report has reported that amino acid residues of 3A4A, including TYR-158, ASP-352, ARG-442, and ARG-315, can interact with ligands [31], which was confirmed in this study.

**Table 2:** The active sites, number of hydrogen bonds, Kd value, the affinity of ligands for 3A4A, and their inhibitory activities on α-glucosidase.

No.	Compound name	Active sites	Number of hydrogen bonds	Affinity (kcal/mol)	Kd (M)	IC_50_ (mM)	Inhibitory (%)
**C1**	5-Hydroxy-3,7,3’,4’-tetramethoxyflavone	ASP-352	1	−8.3	5.54E-11	–	24.34^b^
**C2**	5-Demethylnobiletin	SER-240	1	−7.5	<1.0E-12	–	26.11^a^
**C3**	Vicenin-3	THR-310, ARG-315	2	−8.4	7.09E-12	0.41	–
**C4**	Naringin	LYS-156, GLU-277, GLN-279, ASP-352	4	−10.2	8.95E-06	–	19.15^c^
**C5**	8-Hydroxy-3,5,6,7,3′,4′-hexamethoxyflavone	LYS-156, ARG-442	2	−8.0	1.00E-05	–	22.78^b^
**C6**	4′,5,6,7-Tetramethoxyflavone	ARG-315, GLU-411, ARG-442	3	−8.2	1.18E-07	–	26.77^b^
**C7**	Tangeretin	HIS-280, ARG-315, ARG-442	4	−8.4	3.99E-05	–	20.54^a^
**C8**	Neohesperidin	LYS-156, ASP-242, GLN-279, ASP-352	5	−10.1	8.95E-06	24.15	–
**C9**	Nobiletin	HIS-280, ARG-315, ARG-442	3	−8.2	8.11E-06	–	18.98^c^
**C10**	Quercetagetin-3,5,6,7,3′,4′-hexamethyl ether	LYS-156	1	−7.9	4.54E-07	–	23.21^b^
**C11**	Sinensetin	GLN-279, ARG-315	2	−7.8	2.40E-06	–	22.00^c^
**C12**	Isosinensetin	LYS-13	1	−8.4	2.41E-06	–	9.74^c^
**C13**	Hesperidin	SER-157, GLU-277, HIS-280, THR-310, ARG-315	5	−10.4	1.00E-07	–	10.97^c^
**C14**	Naringenin	TYR-158	1	−8.2	5.72E-06	0.40	–
**C15**	Naringenin-7-O-glucoside	LYS-156, GLU-277, GLN-353, GLU-411	4	−10.2	2.94E-06	8.90	–
**C16**	Eriocitrin	ASP-69, TYR-158, ASP-215, GLU-277, GLN-353	6	−10.8	3.62E-06	6.71	–
**C17**	Vicenin-2	LYS-13, LYS-16, ASN-259	3	−8.8	3.32E-05	0.43	–
**C18**	Rhoifolin	ASP-215, ASP-307, ARG-315	4	−10.9	1.58E-05	5.28	–
**C19**	Hesperetin	ASP-242, ASN-415	3	−8.7	3.54E-06	0.32	–
**C20**	Isoquercitroside	SER-157, PRO-312, GLN-353, GLU-411, ASN-415	5	−9.3	3.45E-06	1.46	–
**C21**	5,4´-Dihydroxy-3,6,7,8,3´-pentamethoxyflavone	ARG-442	1	−7.8	–	–	–
**C30**	2´-Hydroxy-3,4,4´,5´,6´-pentamethoxychalcone	TYR-158, ARG-442	2	−8.0	–	–	–
**C39**	Quercetagetin-3,7,3′,4′-tetramethyl ether	GLU-277, ASP-352	3	−8.4	–	–	–
**C57**	3,3′,4′,5,6,7,8-heptamethoxyflavone	SER-240, ARG-315	2	−7.6	–	–	–
**C60**	Kaempferol-3-O-rutinoside	ASP-307, PRO-312, ARG-315, ARG-442	4	−10.6	4.55E-06	2.91	–
**C78**	Dihydroxy-trimethoxyflavone	TYR-158, ARG-442	2	−8.0	–	–	–
	Acarbose	LYS-156, TYR-158, ASP-242, HIS-280, SER-304, ASP-307, THR-310, LEU-313	10	−8.6	–	6.41	–

^a^The inhibitory (%) at the concentration of 2.5 mM.

^b^The inhibitory (%) at the concentration of 5 mM.

^c^The inhibitory (%) at the concentration of 10 mM.

The affinity values of the screened compounds with 3A4A were shown in Table 2. From Table 2, we found that the affinity values of **C4**, **C8**, **C13**, **C15**, **C16**, **C18**, **C20**, and **C60** were in the range of −9.3 ~ −10.9 kcal/mol, indicating they had a strong affinity with 3A4A. By observing their structures (shown in Fig 3), we found that the eight compounds contained sugar units such as glucose (Glc) and rhamnose (Rha), indicating that the presence of sugar units in the compound structure could increase the affinity. Except for the eight compounds mentioned above, the other compounds also had relatively weaker inhibitory activities against 3A4A, with affinity values of −7.5 ~ −8.8 kcal/mol, probably because most of them contains no sugar units in their structures.

### Molecular dynamics simulation results

**Fig 6:**
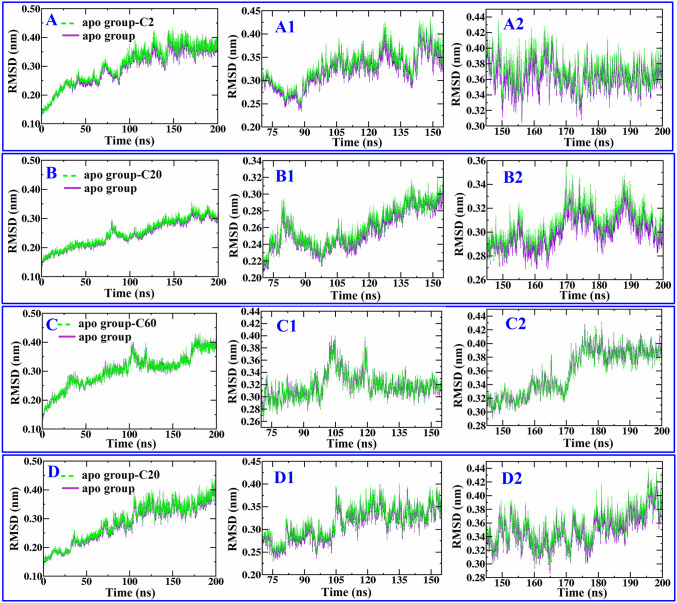
The RMSD of molecular dynamics simulations. **C2**－3A4A complex (A) and its enlarged image (A1, A2), **C20**－3A4A complex (B) and its enlarged image (B1, B2), **C60**－3A4A complex (C) and its enlarged image (C1, C2), acarbose－3A4A complex (D) and its enlarged image (D1, D2).

**Fig 7:**
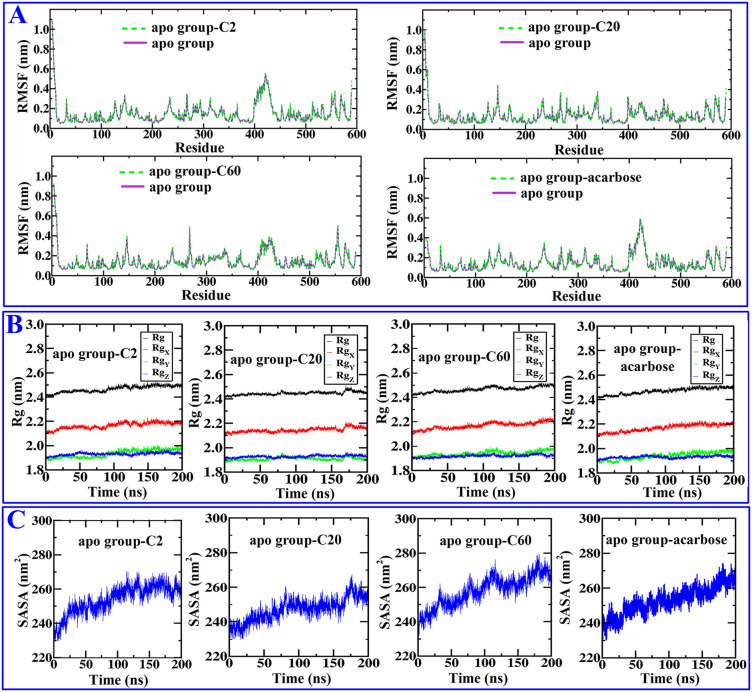
The RMSF (A), Rg (B), and SASA (C) of molecular dynamics simulations.

**Fig 8:**
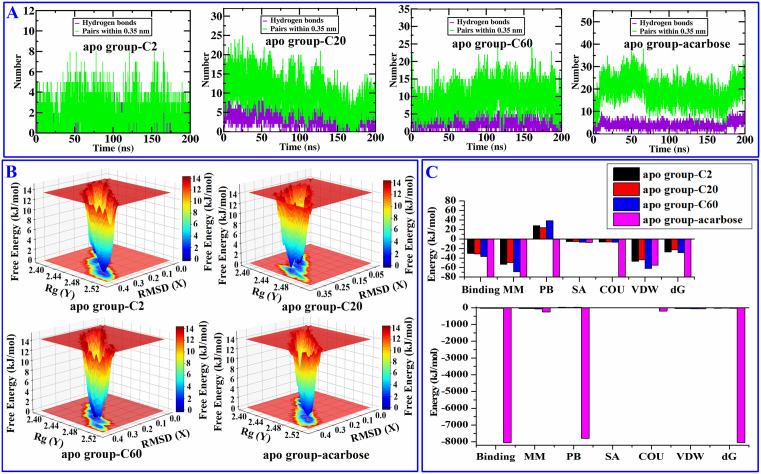
The H-bonds number (A), the distribution of free energy (B), and free energy contribution (C) of molecular dynamics simulations.

From the affinity values of the selected compounds to 3A4A, compounds with relatively lower affinity **C2** (−7.5 kcal/mol), moderate affinity **C20** (−9.3 kcal/mol), and higher affinity **C60** (−10.6 kcal/mol), along with acarbose (−8.6 kcal/mol), were subjected to molecular dynamics simulations to investigate the stability of their binding. The dynamic stability and structural changes of the complex were analyzed using RMSD, RMSF, Rg, SASA, the number of hydrogen bonds between 3A4A and ligands, and the distribution of free energy. The results showed that their RMSD values first increased within the first few nanoseconds of simulation due to the system not reaching equilibrium, reflecting solvent adjustment, local structural relaxation, or conformational rearrangement caused by small molecule binding, and then their RMSD values (after 75 ns) stabilized at 0.20 nm ~ 0.45 nm (shown in Fig 6), especially after 150 ns, their RMSD values all remain within the range of 0.26 nm ~ 0.45 nm, indicating that the fluctuation range further narrowed. This results suggests that the conformation of the four tested complexes were stable. After 75 ns, compound **C20** exhibits the smallest RMSD fluctuation range (≤0.14 nm), followed by **C60** (≤0.19 nm). Both **C2** and acarbose show comparable values, with their RMSD fluctuations remaining within 0.225 nm. This indicates that compound **C20** binds to 3A4A with the highest stability, followed by **C60**, whereas compound **C2** and acarbose exhibit comparable binding stability to 3A4A. The RMSF values for the key residues (LYS-13, LYS-156, SER-157, TYR-158, ASP-215, SER-240, ASP-242, GLU-277, GLN-279, HIS-280, ASP-307, THR-310, PRO-312, ARG-315,ASP-352, GLN-353, GLU-411, ASN-415, ARG-442) involved in the interactions between the four tested compounds and 3A4A consistently remained at a low level, with no significant fluctuations observed. This indicates that the four tested compounds formed stable interactions with the key residues of 3A4A (shown in Fig 7A). The Rg values of the four tested complexes are all around 2.45 nm with minor fluctuations (shown in Fig 7B), indicating the tested four compounds separately formed stable and compact complex with 3A4A. The SASA values reflect the degree of exposure of ligands to solvents, Fig 7C shows both **C2**－3A4A and **C20**－3A4A stabilize at 230 ~ 270 nm^2^, whereas **C20**－3A4A and acarbose－3A4A stabilize at 230 ~ 280 nm^2^, indicating that compared to acarbose and **C20**, **C2** and **C20** are enveloped more tightly. Fig 8A shows the hydrogen bond interactions between 3A4A and the four compounds, and the results suggest that throughout the simulation process, the four tested compounds maintained continuous hydrogen bonding with the 3A4A. In Fig 8B, energy troughs (dark blue area) correspond to high probability conformational clusters, and each energy trough is corresponding to low RMSD value (< 0.2 nm) and compact Rg value (< 0.1 nm), indicating each complex is in a preferred conformation. The free bounding energies (Binding) of compounds **C2**, **C20**, **C60** as well as acarbose are −30.30 kJ/mol, −31.28 kJ/mol, −36.80 kJ/mol, and −8053.67 kJ/mol, respectively, indicating in the process of dynamic simulation, acarbose binds more stably to 3A4A. For compound **C2**, **C20** as well as **C60**, van der Waals (VDW) and the average molecular mechanical energy (MM) contribute the most to the free energy, followed by Gibbs free energy (dG), whereas for acarbose, dG contribute the most to the free energy, followed by Poisson Bolzmann (PB) (shown in Fig 8C). The above results indicate that **C2**, **C20**, and **C60**, which exhibit varying degrees of affinity for 3A4A, all form stable complexes with it in molecular dynamics simulations. These findings suggest that the other screened compounds also bind stably to 3A4A, further indicating that they probably are inhibitors of α-glucosidase.

### The Kd value and *in vitro* enzyme inhibition assay

The Kd value was detected using each pure compound, and the results (shown in Table 2) suggested that compound **C1** (5-hydroxy-3,7,3’,4’-tetramethoxyflavone), **C2** (5-demethylnobiletin), and **C3** (vicenin-3) had strong affinities with α-glucosidase, whereas the other detected compounds had medium affinities with α-glucosidase. In order to further verify the α-glucosidase inhibitory activity of the selected ligands, *in vitro* enzyme inhibition assay was performed. Due to the reference standards of **C1**–**C20** as well as **C60** can be commercially obtained, we conducted the *in vitro* enzyme inhibition assay on these compounds, and the results were shown in Table 2. From Table 2, we found that these tested compounds had significant differences in the inhibition degree on α-glucosidase. The four compounds vicenin-3 (**C3**), naringenin (**C14**), vicenin-2 (**C17**) and hesperetin (**C19**) had strong inhibitory activity on α-glucosidase, and their IC_50_ values were 0.41 mM, 0.40 mM, 0.43 mM as well as 0.32 mM, respectively. Isoquercitroside (**C20**) and kaempferol-3-O-rutinoside (**C60**) exhibited weaker inhibitory activity against α-glucosidase compared to the four compounds mentioned above, with IC_50_ values of 1.46 mM and 2.91 mM, respectively. Although the compound rhoifolin (**C18**, IC_50_ 5.28 mM) had a relatively weak inhibitory activity on α-glucosidase, it was still stronger than the positive control drug acarbose (IC_50_ 6.41 mM). However, the remaining fourteen compounds showed weaker inhibitory activity on α-glucosidase.

In this study, total 84 α-glucosidase inhibitors were identified in *Citri Reticulatae Pericarpium*, and among them, only 13 compounds, including vicenin-3 (**C3**), naringin (**C4**), tangeretin (**C7**), neohesperidin (**C8**), sinensetin (**C11**), hesperidin (**C13**), naringenin (**C14**), diosmetin (**C16**), vicenin-2 (**C17**), rhoifolin (**C18**), hesperetin (**C19**), isoquercitroside (**C20**), and kaempferol-3-O-rutinoside (**C60**), had been previously reported to exhibit inhibitory activity against α-glucosidase [32–39]. The remaining 71 compounds were first reported as α-glucosidase inhibitors in this study, and most of them were isomers of the known compounds.

It is worth noting that quite often a compound is shown with a strong binding affinity with a protein in molecular docking and *in vitro* experiments but failed to show any activity *in vivo*, because the action of a compound on a particular protein is complex and depends on many factors, including concentrations and bioavailability of the compound *in vivo*. Findings from our study are required for further experimental validation.

In addition, through carefully observed the screened ligands with definite structures (shown in Fig 3), it was found that most of the screened ligands were multimethoxy flavonoids, which were a type of compounds unique to the citrus genus. Modern pharmacological research have showed that the multimethoxy flavonoids in *Citrus* have significant anti-inflammatory, anti-tumor, neuroprotective, fat reducing as well as weight reducing, anti-lipase activity, digestion promoting, anti-virus, and anti-oxidant activities [40]. Our study first reported that multimethoxy flavonoids could inhibit the activity of α-glucosidase, probably indicating they had potential value of anti-diabetes.

## Conclusions

This study systematically screened α-glucosidase inhibitors from *Citri Reticulatae Pericarpium* using affinity ultrafiltration technology combined with UPLC-ESI-Orbitrap-MS technology for the first time. Compared with traditional methods for screening active ingredients from traditional Chinese medicine, our study has the advantages of small workload, short experimental cycle, and can achieve high-throughput screening capabilities. The molecular docking showed that the compounds with well-defined structures had affinities with α-glucosidase, and molecular dynamics simulation indicated that the ligands bound relatively stable with α-glucosidase. In addition, *in vitro* enzyme inhibition activity experiments suggested that they could inhibit α-glucosidase activity. This study indicated that inhibiting the activity of α-glucosidase may be one of the most important mechanisms for *Citri* *Reticulatae Pericarpium* exerting its hypoglycemic effect, and the active ingredients were mainly flavonoids, especially multimethoxy flavonoids and likely responsible for the observed effects.

## Supporting information

S1 Table The data of affinity ultrafiltration screening and only compounds with PAR value>1 as well as *p*<0.05 (n = 4) were kept. (XLSX)

S2 Table The fragmentation ions of α-glucosidase inhibitors selected from *Citri Reticulatae Pericarpium* extract. (DOCX)
