# Supplementary material for: Global profiling of α-glucosidase inhibitors from Citri Reticulatae Pericarpium based on affinity ultrafiltration screening coupled with UPLC-ESI-Orbitrap-MS method
Source: PLoS One. 2026 Jan 13;21(1):e0340990. doi: 10.1371/journal.pone.0340990 (PMC12798990; doi:10.1371/journal.pone.0340990)
Supplement: S2 Table — (DOCX) [file pone.0340990.s002.docx]

**Global profiling of α-glucosidase inhibitors from *Citri Reticulatae Pericarpium* based on affinity ultrafiltration screening coupled with UPLC-ESI-Orbitrap-MS method**

Hongping Wang^1^, Qiong Yin^1^, Zhaozhou Lin^2^, Quantao Ma^1^, Zhaohua Zhang^1*^, Jun Jia^1*^

^1^ Department of Drug Screening and Development, Scientific Research Institute of Beijing Tongrentang Co., Ltd., Beijing, China

^2^ Department of Drug Screening and Development, Beijing Tongrentang Technology Development Co., Ltd., Beijing, China

^*^ Corresponding author

E-mail: [tongrentangsci@163.com](mailto:tongrentangsci@163.com) (Zhaohua Zhang); [jiajun@tongrentang.com](mailto:jiajun@tongrentang.com) (Jun Jia)

**Supporting Information**

**S2. Table The fragmentation ions of α-glucosidase inhibitors selected from *Citri Reticulatae Pericarpium* extract.**

| **No.** | ***t*_R_ (min)** | **Molecular formula** | **Obtained molecular weight** | **Compound name** | **Product ions** |
| --- | --- | --- | --- | --- | --- |
| **C1** | 25.09 | C_19_H_18_O_7_ | 358.1052 | 5-Hydroxy-3,7,3',4'-tetramethoxyflavone | 344.0894[M+H-CH_3_]^+^,  329.0659[M+H-2CH_3_]^+^,  326.0786[M+H-CH_3_-H_2_O]^+^,  298.0828[M+H-CH_3_-H_2_O-CO]^+^,  283.0607[M+H-2CH_3_-H_2_O-CO]^+^ |
| **C2** | 23.81 | C_20_H_20_O_8_ | 388.1157 | 5-Demethylnobiletin | 374.0998[M+H-CH_3_]^+^,  359.0762[M+H-2CH_3_]^+^,  356.0899[M+H-CH_3_-H_2_O]^+^,  341.0655[M+H-2CH_3_-H_2_O]^+^,  331.0815[M+H-2CH_3_-CO]^+^,  313.0699[M+H-2CH_3_-H_2_O-CO]^+^ |
| **C3** | 7.98 | C_26_H_28_O_14_ | 564.1478 | Vicenin-3 | 433.1112[M+H-Ara]^+^,  415.1034[M+H-Ara-H_2_O]^+^,  397.0914[M+H-Ara-2H_2_O]^+^,  379.0835[M+H-Ara-3H_2_O]^+^,  367.0822[M+H-Ara-2H_2_O-OCH_2_]^+^ |
| **C4** | 9.92 | C_27_H_32_O_14_ | 580.1789 | Naringin | 273.0767[M+H-Rha-Glc]^+^ |
| **C5** | 24.62 | C_21_H_22_O_9_ | 418.1263 | 8-Hydroxy-3,5,6,7,3′,4′-hexamethoxyflavone | 404.1100[M+H-CH_3_]^+^,  389.0865[M+H-2CH_3_]^+^,  386.1001[M+H-CH_3_-H_2_O]^+^,  371.0760[M+H-2CH_3_-H_2_O]^+^,  361.0919[M+H-2CH_3_-CO]^+^,  346.0681[M+H-3CH_3_-CO]^+^,  328.0575[M+H-3CH_3_-CO-H_2_O]^+^,  313.0349[M+H-4CH_3_-CO-H_2_O]^+^ |
| **C6** | 24.93 | C_19_H_18_O_6_ | 342.1102 | 4′,5,6,7-Tetramethoxyflavone | 328.0934[M+H-CH_3_]^+^,  313.0706[M+H-2CH_3_]^+^,  299.0913[M+H-CH_3_-CHO]^+^,  285.0752[M+H-2CH_3_-CO]^+^,  257.0790[M+H-2CH_3_-2CO]^+^ |
| **C7** | 22.82 | C_20_H_20_O_7_ | 372.1206 | Tangeretin | 358.1049[M+H-CH_3_]^+^,  343.0814[M+H-2CH_3_]^+^,  315.0857[M+H-2CH_3_-CO]^+^ |
| **C8** | 10.73 | C_28_H_34_O_15_ | 610.1903 | Neohesperidin | 303.0861[M+H-Rha-Glc]^+^ |
| **C9** | 20.73 | C_21_H_22_O_8_ | 402.1311 | Nobiletin | 388.1148[M+H-CH_3_]^+^,  373.0912[M+H-2CH_3_]^+^,  355.0807[M+H-2CH_3_-H_2_O]^+^,  327.0864[M+H-2CH_3_-H_2_O-CO]^+^ |
| **C10** | 20.00 | C_21_H_22_O_8_ | 402.1313 | Quercetagetin-3,5,6,7,3′,4′-hexamethyl ether | 388.1153[M+H-CH_3_]^+^,  373.0919[M+H-2CH_3_]^+^,  355.0817[M+H-2CH_3_-H_2_O]^+^,  327.0862[M+H-2CH_3_-H_2_O-CO]^+^ |
| **C11** | 18.69 | C_20_H_20_O_7_ | 372.1207 | Sinensetin | 358.1044[M+H-CH_3_]^+^,  343.0815[M+H-2CH_3_]^+^,  315.0876[M+H-2CH_3_-CO]^+^ |
| **C12** | 17.05 | C_20_H_20_O_7_ | 372.1207 | Isosinensetin | 358.1049[M+H-CH_3_]^+^,  343.0813[M+H-2CH_3_]^+^,  315.0867[M+H-2CH_3_-CO]^+^ |
| **C13** | 10.32 | C_28_H_34_O_15_ | 610.1894 | Hesperidin | 303.0861[M+H-Rha-Glc]^+^ |
| **C14** | 10.03 | C_15_H_12_O_5_ | 272.0685 | Naringenin | 179.0342[M+H-C_6_H_6_O]^+^,  153.0183[M+H-C_8_H_8_O]^+^ |
| **C15** | 10.06 | C_21_H_22_O_10_ | 434.1213 | Naringenin-7-O-glucoside | 273.0744[M+H-Glc]^+^ |
| **C16** | 8.55 | C_27_H_32_O_15_ | 596.1741 | Eriocitrin | 289.0708[M+H-Rha-Glc]^+^ |
| **C17** | 6.86 | C_27_H_30_O_15_ | 594.1584 | Vicenin-2 | 541.1353[M+H-3H_2_O]^+^,  523.1260[M+H-4H_2_O]^+^,  433.1002[M+H-Glc]^+^,  271.0600[M+H-2Glc]^+^ |
| **C18** | 9.58 | C_27_H_30_O_14_ | 578.1635 | Rhoifolin | 415.1093[M+H-Rha-H_2_O]^+^,  397.0886[M+H-Rha-2H_2_O]^+^,  379.0814[M+H-Rha-3H_2_O]^+^,  271.0591[M+H-Rha-Glc]^+^ |
| **C19** | 15.57 | C_16_H_14_O_6_ | 302.0790 | Hesperetin | 285.0752[M+H-H_2_O]^+^,  153.0182[M+H-C_9_H_10_O]^+^ |
| **C20** | 8.72 | C_21_H_20_O_12_ | 464.0954 | Isoquercitroside | 303.0500[M+H-Glc]^+^,  285.0385[M+H-Glc-H_2_O]^+^,  257.0445[M+H-Glc(f)-H_2_O-CO]^+^,  229.0499[M+H-Glc(f)-H_2_O-2CO]^+^,  201.0555[M+H-Glc(f)-H_2_O-3CO]^+^ |
| **C21** | 23.72 | C_20_H_20_O_9_ | 404.1107 | 5,4´-Dihydroxy-3,6,7,8,3´-pentamethoxyflavone | 390.0941[M+H-CH_3_]^+^,  375.0710[M+H-2CH_3_]^+^,  372.0835[M+H-CH_3_-H_2_O]^+^,  357.0606[M+H-2CH_3_-H_2_O]^+^,  347.0761[M+H-2CH_3_-CO]^+^,  332.0533[M+H-3CH_3_-CO]^+^,  329.0666[M+H-2CH_3_-H_2_O-CO]^+^,  317.0286[M+H-4CH_3_-CO]^+^,  304.0592[M+H-3CH_3_-2CO]^+^ |
| **C22** | 6.46 | C_27_H_32_O_15_ | 596.1741 | Eriocitrin isomer | 289.0705[M+H-Rha-Glc]^+^ |
| **C23** | 12.61 | C_19_H_18_O_7_ | 358.1047 | 5-Hydroxy-3,7,3',4'-tetramethoxyflavone isomer | 344.0876[M+H-CH_3_]^+^,  329.0656[M+H-2CH_3_]^+^ |
| **C24** | 24.16 | C_20_H_20_O_8_ | 388.1157 | 5-Demethylnobiletin isomer | 374.0996[M+H-CH_3_]^+^,  359.0760[M+H-2CH_3_]^+^,  356.0806[M+H-CH_3_-H_2_O]^+^,  341.0646[M+H-2CH_3_-H_2_O]^+^,  331.0814[M+H-2CH_3_-CO]^+^,  313.0705[M+H-2CH_3_-H_2_O-CO]^+^ |
| **C25** | 23.19 | C_19_H_18_O_7_ | 358.1053 | 5-Hydroxy-3,7,3',4'-tetramethoxyflavone isomer | 344.0881[M+H-CH_3_]^+^,  329.0646[M+H-2CH_3_]^+^,  326.0787[M+H-CH_3_-H_2_O]^+^,  298.0842[M+H-CH_3_-H_2_O-CO]^+^,  283.0596[M+H-2CH_3_-H_2_O-CO]^+^ |
| **C26** | 23.03 | [C](https://pubchem.ncbi.nlm.nih.gov/#query=C20H22O7)_[20](https://pubchem.ncbi.nlm.nih.gov/#query=C20H22O7)_[H](https://pubchem.ncbi.nlm.nih.gov/#query=C20H22O7)_[22](https://pubchem.ncbi.nlm.nih.gov/#query=C20H22O7)_[O](https://pubchem.ncbi.nlm.nih.gov/#query=C20H22O7)_[7](https://pubchem.ncbi.nlm.nih.gov/#query=C20H22O7)_ | 374.1368 | 2´-Hydroxy-3,4,4´,5´,6´-pentamethoxychalcone isomer | 211.0602[M+H-C_10_H_12_O_2_]^+^,  196.0365[M+H-C_10_H_12_O_2_-H_2_O]^+^,  191.0704[M+H-C_10_H_12_O_2_-OCH_2_]^+^,  150.0318[M+H-C_10_H_12_O_2_-2H_2_O-CO]^+^ |
| **C27** | 16.18 | [C](https://pubchem.ncbi.nlm.nih.gov/#query=C20H22O7)_[20](https://pubchem.ncbi.nlm.nih.gov/#query=C20H22O7)_[H](https://pubchem.ncbi.nlm.nih.gov/#query=C20H22O7)_[22](https://pubchem.ncbi.nlm.nih.gov/#query=C20H22O7)_[O](https://pubchem.ncbi.nlm.nih.gov/#query=C20H22O7)_[7](https://pubchem.ncbi.nlm.nih.gov/#query=C20H22O7)_ | 374.1365 | 2´-Hydroxy-3,4,4´,5´,6´-pentamethoxychalcone isomer | 211.0600[M+H-C_10_H_12_O_2_]^+^,  196.0365[M+H-C_10_H_12_O_2_-H_2_O]^+^,  191.0702[M+H-C_10_H_12_O_2_-OCH_2_]^+^,  168.0417[M+H-C_10_H_12_O_2_-H_2_O-CO]^+^,  150.0311[M+H-C_10_H_12_O_2_-2H_2_O-CO]^+^ |
| **C28** | 9.93 | C_28_H_34_O_15_ | 610.1903 | Neohesperidin isomer/Hesperidin isomer | 303.0865[M+H-Rha-Glc]^+^ |
| **C29** | 22.32 | C_19_H_18_O_7_ | 358.1053 | 5-Hydroxy-3,7,3',4'-tetramethoxyflavone isomer | 344.0890[M+H-CH_3_]^+^,  329.0651[M+H-2CH_3_]^+^,  326.0787[M+H-CH_3_-H_2_O]^+^,  298.0836[M+H-CH_3_-H_2_O-CO]^+^,  283.0591[M+H-2CH_3_-H_2_O-CO]^+^ |
| **C30** | 19.38 | [C](https://pubchem.ncbi.nlm.nih.gov/#query=C20H22O7)_[20](https://pubchem.ncbi.nlm.nih.gov/#query=C20H22O7)_[H](https://pubchem.ncbi.nlm.nih.gov/#query=C20H22O7)_[22](https://pubchem.ncbi.nlm.nih.gov/#query=C20H22O7)_[O](https://pubchem.ncbi.nlm.nih.gov/#query=C20H22O7)_[7](https://pubchem.ncbi.nlm.nih.gov/#query=C20H22O7)_ | 374.1366 | 2´-Hydroxy-3,4,4´,5´,6´-pentamethoxychalcone | 211.0600[M+H-C_10_H_12_O_2_]^+^,  196.0365[M+H-C_10_H_12_O_2_-H_2_O]^+^,  191.0706[M+H-C_10_H_12_O_2_-OCH_2_]^+^,  168.0417[M+H-C_10_H_12_O_2_-H_2_O-CO]^+^,  150.0312[M+H-C_10_H_12_O_2_-2H_2_O-CO]^+^ |
| **C31** | 7.18 | C_15_H_12_O_5_ | 272.0684 | Naringenin isomer | 179.0341[M+H-C_6_H_6_O]^+^,  153.0192[M+H-C_8_H_8_O]^+^ |
| **C32** | 8.43 | C_21_H_20_O_12_ | 464.0955 | Isoquercitroside isomer | 303.0500[M+H-Glc]^+^,  285.0395[M+H-Glc-H_2_O]^+^,  257.0437[M+H-Glc-H_2_O-CO]^+^,  229.0497[M+H-Glc-H_2_O-2CO]^+^,  201.0542[M+H-Glc-H_2_O-3CO]^+^ |
| **C33** | 8.55 | C_21_H_22_O_10_ | 434.1213 | Naringenin-7-O-glucoside isomer | 273.0760[M+H-Glc]^+^ |
| **C34** | 10.17 | C_15_H_12_O_5_ | 272.0684 | Naringenin isomer | 179.0343[M+H-C_6_H_6_O]^+^,  153.0182[M+H-C_8_H_8_O]^+^ |
| **C35** | 14.16 | C_21_H_22_O_9_ | 418.1264 | 8-Hydroxy-3,5,6,7,3′,4′-hexamethoxyflavone isomer | 404.1096[M+H-CH_3_]^+^,  389.0861[M+H-2CH_3_]^+^,  386.0992[M+H-CH_3_-H_2_O]^+^,  371.0759[M+H-2CH_3_-H_2_O]^+^,  361.0915[M+H-2CH_3_-CO]^+^,  346.0680[M+H-3CH_3_-CO]^+^,  328.0571[M+H-3CH_3_-CO-H_2_O]^+^,  313.0333[M+H-4CH_3_-CO-H_2_O]^+^ |
| **C36** | 9.10 | C_27_H_30_O_14_ | 578.0000 | Rhoifolin isomer | 271.0601[M+H-Rha-Glc]^+^ |
| **C37** | 23.48 | C_20_H_20_O_8_ | 388.1157 | 5-Demethylnobiletin isomer | 374.1002[M+H-CH_3_]^+^,  359.0767[M+H-2CH_3_]^+^,  356.0904[M+H-CH_3_-H_2_O]^+^,  341.0651[M+H-2CH_3_-H_2_O]^+^,  331.0808[M+H-2CH_3_-CO]^+^,  313.0703[M+H-2CH_3_-H_2_O-CO]^+^ |
| **C38** | 10.72 | C_16_H_14_O_6_ | 302.0790 | Hesperetin isomer | 285.0785[M+H-H_2_O]^+^,  153.0182[M+H-C_9_H_10_O]^+^ |
| **C39** | 21.06 | C_19_H_18_O_8_ | 374.1001 | Quercetagetin-3,7,3′,4′-tetramethyl ether | 360.0838[M+H-CH_3_]^+^,  345.0605[M+H-2CH_3_]^+^,  330.0365[M+H-3CH_3_]^+^,  327.0499[M+H-2CH_3_-H_2_O]^+^,  317.0662[M+H-2CH_3_-CO]^+^,  302.0398[M+H-3CH_3_-CO]^+^,  299.0534[M+H-2CH_3_-CO-H_2_O]^+^,  271.0598[M+H-2CH_3_-CO-2H_2_O]^+^ |
| **C40** | 16.79 | C_20_H_20_O_8_ | 388.1158 | 5-Demethylnobiletin isomer | 374.1000[M+H-CH_3_]^+^,  359.0765[M+H-2CH_3_]^+^,  356.0906[M+H-CH_3_-H_2_O]^+^,  341.0654[M+H-2CH_3_-H_2_O]^+^,  331.0809[M+H-2CH_3_-CO]^+^,  313.0705[M+H-2CH_3_-H_2_O-CO]^+^ |
| **C41** | 19.03 | C_18_H_16_O_7_ | 344.0896 | Dihydroxy-trimethoxyflavone isomer | 330.0726[M+H-CH_3_]^+^,  315.0493[M+H-2CH_3_]^+^ |
| **C42** | 8.41 | C_27_H_30_O_14_ | 578.0000 | Rhoifolin isomer | 415.1021[M+H-Rha-H_2_O]^+^,  397.0919[M+H-Rha-2H_2_O]^+^,  379.0817[M+H-Rha-3H_2_O]^+^,  271.0603[M+H-Rha-Glc]^+^ |
| **C43** | 15.81 | C_21_H_22_O_9_ | 418.1264 | 8-Hydroxy-3,5,6,7,3′,4′-hexamethoxyflavone isomer | 404.1104[M+H-CH_3_]^+^,  389.0867[M+H-2CH_3_]^+^,  386.0997[M+H-CH_3_-H_2_O]^+^,  371.0760[M+H-2CH_3_-H_2_O]^+^,  361.0916[M+H-2CH_3_-CO]^+^,  346.0679[M+H-3CH_3_-CO]^+^,  328.0573[M+H-3CH_3_-CO-H_2_O]^+^,  313.0349[M+H-4CH_3_-CO-H_2_O]^+^ |
| **C44** | 8.41 | C_26_H_28_O_14_ | 564.1478 | Vicenin-3 isomer | 433.1134[M+H-Ara]^+^,  415.1025[M+H-Ara-H_2_O]^+^,  397.0922[M+H-Ara-2H_2_O]^+^,  379.0814[M+H-Ara-3H_2_O]^+^,  367.0821[M+H-Ara-2H_2_O-OCH_2_]^+^,  271.0610[M+H-Ara-Glc]^+^ |
| **C45** | 8.51 | C_15_H_12_O_5_ | 272.0684 | Naringenin isomer | 179.0341[M+H-C_6_H_6_O]^+^,  153.0183[M+H-C_8_H_8_O]^+^ |
| **C46** | 22.39 | C_20_H_20_O_9_ | 404.1107 | 5,4´-Dihydroxy-3,6,7,8,3´-pentamethoxyflavone isomer | 390.0935[M+H-CH_3_]^+^,  375.0712[M+H-2CH_3_]^+^,  372.0829[M+H-CH_3_-H_2_O]^+^,  357.0604[M+H-2CH_3_-H_2_O]^+^,  347.0767[M+H-2CH_3_-CO]^+^,  332.0526[M+H-3CH_3_-CO]^+^,  329.0659[M+H-2CH_3_-H_2_O-CO]^+^,  317.0283[M+H-4CH_3_-CO]^+^,  304.0421[M+H-3CH_3_-2CO]^+^,  301.0705[M+H-2CH_3_-H_2_O-2CO]^+^,  289.0359[M+H-4CH_3_-2CO]^+^ |
| **C47** | 9.26 | C_27_H_30_O_15_ | 594.1585 | [Kaempferol-3-O-rutinoside isomer](https://www.chemsrc.com/cas/17297-56-2_89274.html) | 449.1115[M+H-Rha]^+^,  287.0552[M+H-Rha-Glc]^+^ |
| **C48** | 22.09 | C_20_H_20_O_9_ | 404.1107 | 5,4´-dihydroxy-3,6,7,8,3´-pentamethoxyflavone isomer | 390.0943[M+H-CH_3_]^+^,  375.0708[M+H-2CH_3_]^+^,  372.0840[M+H-CH_3_-H_2_O]^+^,  357.0606[M+H-2CH_3_-H_2_O]^+^,  347.0757[M+H-2CH_3_-CO]^+^,  332.0527[M+H-3CH_3_-CO]^+^,  329.0664[M+H-2CH_3_-H_2_O-CO]^+^,  317.0283[M+H-4CH_3_-CO]^+^,  304.0592[M+H-3CH_3_-2CO]^+^,  301.0703[M+H-2CH_3_-H_2_O-2CO]^+^,  289.0359[M+H-4CH_3_-2CO]^+^ |
| **C49** | 20.41 | C_18_H_16_O_7_ | 344.0896 | Dihydroxy-trimethoxyflavone isomer | 330.0739[M+H-CH_3_]^+^,  315.0501[M+H-2CH_3_]^+^,  287.0547[M+H-2CH_3_-CO]^+^,  269.0444[M+H-2CH_3_-CO-H_2_O]^+^ |
| **C50** | 8.26 | C_28_H_34_O_15_ | 610.1899 | Neohesperidin isomer/Hesperidin isomer | 303.0866[M+H-Rha-Glc]^+^ |
| **C51** | 15.97 | C_20_H_20_O_8_ | 388.1157 | 5-Demethylnobiletin isomer | 374.0985[M+H-CH_3_]^+^,  359.0764[M+H-2CH_3_]^+^,  356.0888[M+H-CH_3_-H_2_O]^+^,  341.0657[M+H-2CH_3_-H_2_O]^+^,  331.0811[M+H-2CH_3_-CO]^+^,  328.0950[M+H-CH_3_-H_2_O-CO]^+^,  313.0711[M+H-2CH_3_-H_2_O-CO]^+^ |
| **C52** | 17.79 | C_21_H_22_O_9_ | 418.1263 | 8-Hydroxy-3,5,6,7,3′,4′-hexamethoxyflavone isomer | 404.1100[M+H-CH_3_]^+^,  389.0865[M+H-2CH_3_]^+^,  386.1002[M+H-CH_3_-H_2_O]^+^,  371.0760[M+H-2CH_3_-H_2_O]^+^,  361.0916[M+H-2CH_3_-CO]^+^,  346.0682[M+H-3CH_3_-CO]^+^,  328.0576[M+H-3CH_3_-CO-H_2_O]^+^,  313.0348[M+H-4CH_3_-CO-H_2_O]^+^ |
| **C53** | 12.02 | C_27_H_30_O_14_ | 578.0000 | Rhoifolin isomer | 271.0588[M+H-Rha-Glc]^+^ |
| **C54** | 7.30 | C_27_H_32_O_15_ | 596.1741 | Eriocitrin isomer | 289.0709[M+H-Rha-Glc]^+^ |
| **C55** | 15.20 | C_27_H_32_O_14_ | 580.1792 | Naringin isomer | 273.0757[M+H-Rha-Glc]^+^ |
| **C56** | 14.97 | C_19_H_18_O_7_ | 358.1052 | 5-Hydroxy-3,7,3',4'-tetramethoxyflavone isomer | 344.0893[M+H-CH_3_]^+^,  329.0657[M+H-2CH_3_]^+^,  326.0787[M+H-CH_3_-H_2_O]^+^,  298.0836[M+H-CH_3_-H_2_O-CO]^+^,  283.0602[M+H-2CH_3_-H_2_O-CO]^+^ |
| **C57** | 22.02 | C_22_H_24_O_9_ | 432.1416 | 3,3′,4′,5,6,7,8-heptamethoxyflavone | 418.1262[M+H-CH_3_]^+^,  403.1025[M+H-2CH_3_]^+^,  400.1153[M+H-CH_3_-H_2_O]^+^,  385.0921[M+H-2CH_3_-H_2_O]^+^ |
| **C58** | 18.59 | C_21_H_22_O_9_ | 418.1263 | 8-Hydroxy-3,5,6,7,3′,4′-hexamethoxyflavone isomer | 404.1099[M+H-CH_3_]^+^,  389.0865[M+H-2CH_3_]^+^,  386.1002[M+H-CH_3_-H_2_O]^+^,  371.0764[M+H-2CH_3_-H_2_O]^+^,  361.0916[M+H-2CH_3_-CO]^+^,  346.0695[M+H-3CH_3_-CO]^+^,  328.0576[M+H-3CH_3_-CO-H_2_O]^+^,  313.0332[M+H-4CH_3_-CO-H_2_O]^+^ |
| **C59** | 23.93 | C_21_H_22_O_8_ | 402.1313 | Quercetagetin-3,5,6,7,3′,4′-hexamethyl ether isomer/Nobiletin isomer | 388.1151[M+H-CH_3_]^+^,  373.0917[M+H-2CH_3_]^+^,  355.0813[M+H-2CH_3_-H_2_O]^+^,  327.0861[M+H-2CH_3_-H_2_O-CO]^+^ |
| **C60** | 8.64 | C_27_H_30_O_15_ | 594.1585 | Kaempferol-3-O-rutinoside | 449.1085[M+H-Rha]^+^,  427.1028[M+H-Rha-H_2_O]^+^,  409.0909[M+H-Rha-2H_2_O]^+^,  397.0925[M+H-Rha-H_2_O-OCH_2_]^+^,  391.0803[M+H-Rha-3H_2_O]^+^,  379.0825[M+H-Rha-2H_2_O-OCH_2_]^+^,  287.0551[M+H-Rha-Glc]^+^ |
| **C61** | 19.33 | C_20_H_20_O_7_ | 372.1210 | Tangeretin isomer/Isosinensetin isomer/Sinensetin isomer | 358.1046[M+H-CH_3_]^+^,  343.0812[M+H-2CH_3_]^+^,  315.0867[M+H-2CH_3_-CO]^+^ |
| **C62** | 8.25 | C_26_H_28_O_14_ | 564.1479 | Vicenin-3 isomer | 415.1011[M+H-Ara-H_2_O]^+^,  397.0971[M+H-Ara-2H_2_O]^+^,  379.0846[M+H-Ara-3H_2_O]^+^,  367.0822[M+H-Ara-2H_2_O-OCH_2_]^+^,  349.0722[M+H-Ara-3H_2_O-OCH_2_]^+^,  271.0595[M+H-Ara-Glc]^+^ |
| **C63** | 8.26 | C_16_H_14_O_6_ | 302.0790 | Hesperetin isomer | 285.0748[M+H-H_2_O]^+^,  153.0183[M+H-C_9_H_10_O]^+^ |
| **C64** | 17.59 | C_20_H_20_O_8_ | 388.1157 | 5-Demethylnobiletin isomer | 374.0993[M+H-CH_3_]^+^,  359.0761[M+H-2CH_3_]^+^,  356.0899[M+H-CH_3_-H_2_O]^+^,  341.0650[M+H-2CH_3_-H_2_O]^+^,  331.0807[M+H-2CH_3_-CO]^+^,  313.0710[M+H-2CH_3_-H_2_O-CO]^+^ |
| **C65** | 19.02 | C_19_H_18_O_7_ | 358.1053 | 5-Hydroxy-3,7,3',4'-tetramethoxyflavone isomer | 344.0893[M+H-CH_3_]^+^,  329.0656[M+H-2CH_3_]^+^ |
| **C66** | 9.56 | C_21_H_22_O_10_ | 434.1212 | Naringenin-7-O-glucoside isomer | 273.0768[M+H-Glc]^+^ |
| **C67** | 20.06 | C_18_H_16_O_7_ | 344.0896 | Dihydroxy-trimethoxyflavone isomer | 330.0723[M+H-CH_3_]^+^,  315.0493[M+H-2CH_3_]^+^,  287.0543[M+H-2CH_3_-CO]^+^,  269.0443[M+H-2CH_3_-CO-H_2_O]^+^ |
| **C68** | 18.19 | C_21_H_22_O_8_ | 402.1313 | Quercetagetin-3,5,6,7,3′,4′-hexamethyl ether isomer/Nobiletin isomer | 388.1149[M+H-CH_3_]^+^,  373.0918[M+H-2CH_3_]^+^,  355.0811[M+H-2CH_3_-H_2_O]^+^,  327.0867[M+H-2CH_3_-H_2_O-CO]^+^ |
| **C69** | 20.70 | C_20_H_20_O_7_ | 372.1240 | Tangeretin isomer/Isosinensetin isomer/Sinensetin isomer | 358.1067[M+H-CH_3_]^+^,  343.0812[M+H-2CH_3_]^+^,  315.0874[M+H-2CH_3_-CO]^+^ |
| **C70** | 16.00 | C_19_H_18_O_7_ | 358.1052 | 5-Hydroxy-3,7,3',4'-tetramethoxyflavone isomer | 344.0887[M+H-CH_3_]^+^,  329.0657[M+H-2CH_3_]^+^,  326.0781[M+H-CH_3_-H_2_O]^+^,  298.0834[M+H-CH_3_-H_2_O-CO]^+^,  283.0593[M+H-2CH_3_-H_2_O-CO]^+^ |
| **C71** | 9.57 | C_27_H_32_O_14_ | 580.1789 | Naringin isomer | 273.0767[M+H-Rha-Glc]^+^ |
| **C72** | 14.14 | C_19_H_18_O_7_ | 358.1053 | 5-Hydroxy-3,7,3',4'-tetramethoxyflavone isomer | 344.0903[M+H-CH_3_]^+^,  329.0663[M+H-2CH_3_]^+^ |
| **C73** | 10.30 | C_27_H_32_O_15_ | 596.1741 | Eriocitrin isomer | 289.0715[M+H-Rha-Glc]^+^ |
| **C74** | 18.15 | C_19_H_18_O_6_ | 342.1103 | 4′,5,6,7-Tetramethoxyflavone isomer | 328.0933[M+H-CH_3_]^+^,  313.0702[M+H-2CH_3_]^+^,  299.0911[M+H-CH_3_-CHO]^+^,  285.0749[M+H-2CH_3_-CO]^+^,  257.0809[M+H-2CH_3_-2CO]^+^ |
| **C75** | 10.32 | C_16_H_14_O_6_ | 302.0790 | Hesperetin isomer | 285.0763[M+H-H_2_O]^+^,  153.0183[M+H-C_9_H_10_O]^+^ |
| **C76** | 17.05 | C_19_H_18_O_7_ | 358.1047 | 5-Hydroxy-3,7,3',4'-tetramethoxyflavone isomer | 344.0914[M+H-CH_3_]^+^,  329.0659[M+H-2CH_3_]^+^,  326.0787[M+H-CH_3_-H_2_O]^+^,  298.0829[M+H-CH_3_-H_2_O-CO]^+^,  283.0598[M+H-2CH_3_-H_2_O-CO]^+^ |
| **C77** | 9.57 | C_15_H_12_O_5_ | 272.0684 | Naringenin isomer | 179.0340[M+H-C_6_H_6_O]^+^,  153.0183[M+H-C_8_H_8_O]^+^ |
| **C78** | 10.31 | C_18_H_16_O_7_ | 344.0896 | Dihydroxy-trimethoxyflavone | 330.0732[M+H-CH_3_]^+^,  315.0490[M+H-2CH_3_]^+^,  287.0546[M+H-2CH_3_-CO]^+^,  269.0445[M+H-2CH_3_-CO-H_2_O]^+^ |
| **C79** | 19.12 | C_19_H_18_O_6_ | 342.1101 | 4′,5,6,7-Tetramethoxyflavone isomer | 328.0935[M+H-CH_3_]^+^,  313.0707[M+H-2CH_3_]^+^,  299.0913[M+H-CH_3_-CHO]^+^,  285.0750[M+H-2CH_3_-CO]^+^,  257.0799[M+H-2CH_3_-2CO]^+^ |
| **C80** | 7.39 | C_27_H_32_O_14_ | 580.1790 | Naringin isomer | 273.0760[M+H-Rha-Glc]^+^ |
| **C81** | 7.39 | C_21_H_22_O_10_ | 434.1213 | Naringenin-7-O-glucoside isomer | 273.0755[M+H-Glc]^+^ |
| **C82** | 14.53 | C_19_H_18_O_7_ | 358.1053 | 5-Hydroxy-3,7,3',4'-tetramethoxyflavone isomer | 344.0888[M+H-CH_3_]^+^,  329.0653[M+H-2CH_3_]^+^,  326.0785[M+H-CH_3_-H_2_O]^+^,  298.0835[M+H-CH_3_-H_2_O-CO]^+^,  283.0602[M+H-2CH_3_-H_2_O-CO]^+^ |
| **C83** | 8.25 | C_15_H_12_O_5_ | 272.0684 | Naringenin isomer | 179.0339[M+H-C_6_H_6_O]^+^,  153.0182[M+H-C_8_H_8_O]^+^ |
| **C84** | 7.39 | C_15_H_12_O_5_ | 272.0685 | Naringenin isomer | 179.0344[M+H-C_6_H_6_O]^+^,  153.0183[M+H-C_8_H_8_O]^+^ |
